# Culture and transfection: Two major bottlenecks in understanding *Plasmodium vivax* biology

**DOI:** 10.3389/fmicb.2023.1144453

**Published:** 2023-04-04

**Authors:** Sanju Kumari, Abhinav Sinha

**Affiliations:** ICMR-National Institute of Malaria Research, New Delhi, India

**Keywords:** *Plasmodium vivax*, reticulocytes, *in vitro* culture, transfection, malaria

## Abstract

The long term *in vitro* culture of *Plasmodium falciparum* was successfully established by Trager and Jensen in 1976; however it largely remains unachieved for *P. vivax*. The major obstacle associated with *Plasmodium vivax in vitro* culture is its predilection for invading younger reticulocytes and the complex remodelling of invaded reticulocytes. There are many factors under exploration for this predilection and host–parasite interactions between merozoites and invaded reticulocytes. These include various factors related to parasite, host and environment such as compromised reticulocyte osmotic stability after invasion, abundance of iron in the reticulocytes which makes them favourable for *P. vivax* growth and propagation and role of a hypoxic environment in *P. vivax in vitro* growth. *P. vivax* blood stage transfection represents another major hurdle towards understanding this parasite’s complex biology. Efforts in making this parasite amenable for molecular investigation by genetic modification are limited. Newer approaches in sustaining a longer *in vitro* culture and thereby help advancing transfection technologies in *P. vivax* are urgently needed that can be explored to understand the unique biology of this parasite.

## Background

*Plasmodium vivax* is the most widespread Plasmodium species, causing the second highest mortality and morbidity from malaria after *Plasmodium falciparum*. According to the World Health Organization (2021), although the proportion of estimated *P. vivax* cases in the world went down from 8% in 2000 to 2% in 2020, *P. vivax* contributes an estimated 36% and 30% of all malaria cases in WHO South East Asia and Western Pacific region, respectively. It remains the dominant species (>50% of local cases) in Bhutan, Korea, Myanmar, Nepal and Thailand in 2020 (World Health Organization, 2021). *Plasmodium vivax* grabbed clinical attention only since last decade due to reports presenting *P. vivax* associated severe malaria and deaths (Kochar et al., 2005, 2009; Baird, 2007; Price et al., 2007; Anstey et al., 2009; Alexandre et al., 2010; Costa et al., 2011; Douglas et al., 2013, 2014; Patriani et al., 2019). *Plasmodium vivax* is also thought to be a major barrier in malaria elimination and eradication, mostly because of its chronic and relapsing symptoms. Despite enormous clinical and public health importance, *P. vivax* has not been able to take hold of research attention it deserves mainly because of the two major bottlenecks in understanding the clinical and basic biology of the parasite-lack of long-term *in vitro* culture of its blood stages and sub-optimal development of transfection technologies for its genetic manipulation. Therefore, this review focuses on latest updates on these two hurdles and suggests potential ways to circumvent them.

## Contextual *plasmodium vivax* biology

**Figure 1:**
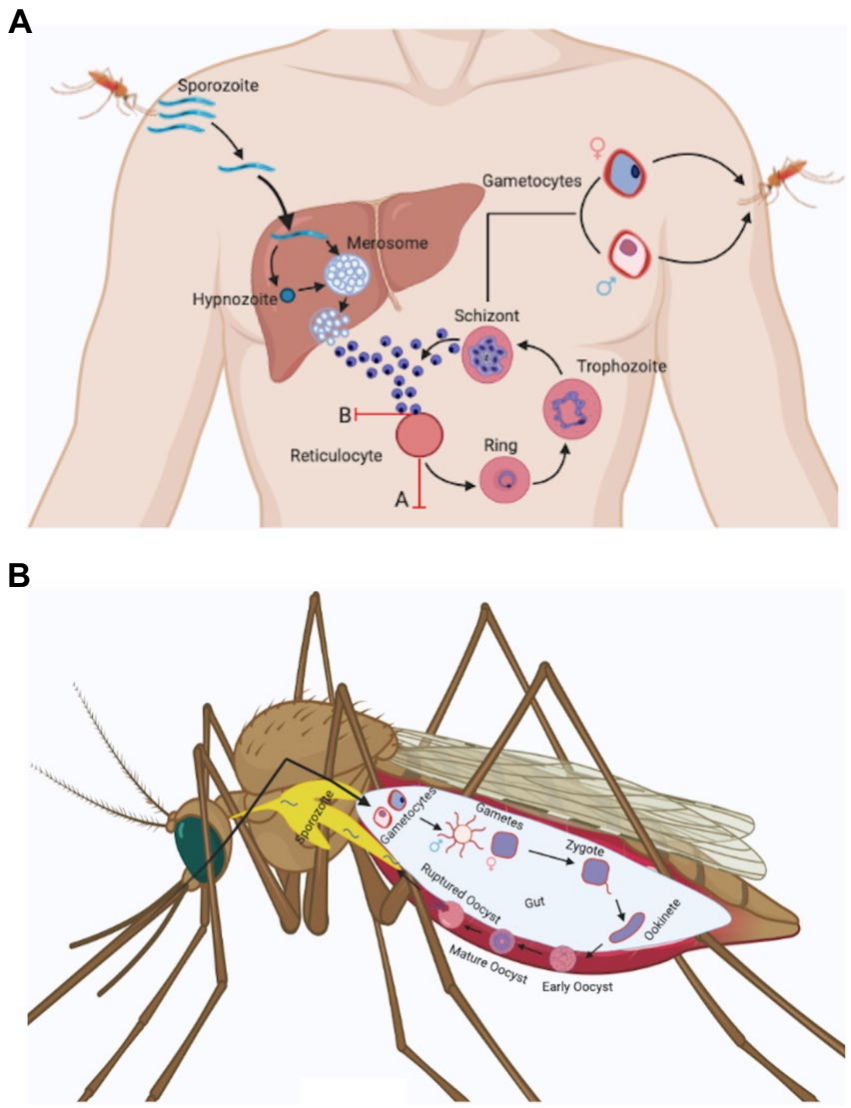
*Plasmodium vivax* life cycle. (a) *Anopheles* spp. mosquito inoculates *Plasmodium* sporozoites into the dermis of human skin that migrate to blood stream and finally invade liver. In the liver, *Plasmodium* sporozoites differentiate either into schizonts or into a dormant form called as hypnozoites that can activate after months or years and release merozoites. *Plasmodium vivax* hepatic merozoites invade reticulocytes in the bloodstream and start the formation of caveola-like complexes that helps in the completion of blood schizogony. Some of the asexual parasites undergo gametocytogenesis and form rounded gametocytes (after 4 days of infection) that are sucked up along with blood meal by another *Anopheles* mosquito (Bourgard et al., 2018). Two major obstacles associated with *Plasmodium vivax in vitro* culture are shown in the figure with red marks. These include: **(A)** Reticulocyte maturation: Reticulocytes mature rapidly *in vitro* condition (Malleret et al., 2015). **(B)** Loss of invasion: *Plasmodium vivax* merozoites lose their ability to invade uninfected reticulocytes *in vitro* after a few cycles (Bermudez et al., 2018). (b) Male and female gametocytes taken up by the mosquito differentiate into male and female gametes and fuse to form zygote in the mosquito mid gut. The zygote differentiates into motile ookinetes which cross the midgut epithelium and divide further to form oocyst. The oocyst ruptures to release hundreds of sporozoites that traverse to salivary glands and can be transmitted to a new host with a fresh mosquito bite (Mueller et al., 2009).

The life cycle and biology of *Plasmodium vivax* (Figure 1) is unlike other Plasmodium species. Its distinguishing features involves: (i) development of sporozoite into a dormant form in hepatocytes called hypnozoites (Cogswell, 1992), (ii) *P. vivax* merozoites’ predilection towards reticulocytes (Field and Shute, 1956) unlike other species of Plasmodium and this possibly results in significantly lower parasitemia, (iii) Presence of spherical shaped gametocytes in the peripheral blood before the appearance of clinical symptoms (Boyd and Kitchen, 1937), (iv) Presence of all blood stage developmental forms (ring, trophozoite, schizont, and gametocytes) in the peripheral blood. However, the mature stages, i.e., trophozoite and schizont are less common in peripheral blood than immature stages (Rudolf and Ramsay, 1927; Field and Shute, 1956; Lopes et al., 2014).

After the bite of an infected female Anopheles mosquitoes, sporozoites move from skin to liver in <30 min, infect hepatocytes and matures to form an actively dividing hepatic schizont or an inactive hypnozoite (Figure 1; Krotoski, 1985). The hypnozoite may remain in quiescent state for months or years and instigates clinical relapse(s) after entering the bloodstream. The cause behind the activation of hypnozoites is not explored, though parasitic and bacterial infection could be a probable factor (Shanks and White, 2013). In coendemic regions where both *P. vivax* and *P. falciparum* exist, a high risk of *P. vivax* parasitaemia was observed after *P. falciparum* treatment (Douglas et al., 2011; Commons et al., 2019). A meta-analysis performed by Commons et al. demonstrated that after 63 days of treatment of *P. falciparum* infection the risk of *P. vivax* recurrence was 24% and it represented almost 70% of all parasitological recurrences. However, the mechanism behind the increased risk of *P. vivax* parasitaemia after treatment of *P. falciparum* infection remains unclear, it has been hypothesised to be due to a possible relapse of *P. vivax* hypnozoites (Hossain et al., 2020).

Another unique feature of *P. vivax* is its predilection for reticulocytes as host cells (Kitchen, 1938). Reticulocytes represent 1%–2% of total red blood cells and this is the probable reason of lower parasitemia in *P. vivax* infected patients. Reticulocytes are larger than erythrocytes (Riley et al., 2001) therefore *P. vivax* infected cells appear larger as compared to uninfected/*P. falciparum*-infected erythrocytes present in peripheral blood smear. *P. vivax* during its development period inside the host cells produces specific proteins which makes cleft like structure in the host cell membrane and caveolae-vesicle complexes which looks like small dots in Giemsa–stained smear called as Schuffner’s dots (Aikawa et al., 1975; Mueller et al., 2009; Akinyi et al., 2012; Gunalan et al., 2020). The biological role of these dots remains largely unexplored but these dots are inconspicuous during *P. vivax* cultures (Gunalan et al., 2020).

One of the unique identifying feature of *P. vivax* is, the amoeboid shape (cytoplasm having finger like projections) of red blood cells infected with this parasite (Suwanarusk et al., 2004). Suwanarusk et al. (2004) experimentally showed that the *P. vivax* infected red blood cells (Pv-iRBCs) increase in deformability so that it can pass through the sinusoid of the spleen easily. The increased deformability of *Pv*-iRBCs may represent a way to avoid its clearance from spleen (Suwanarusk et al., 2004; Handayani et al., 2009). Further, this may lead to the passage of all parasite stages in the peripheral blood (Suwanarusk et al., 2004). Presence of all stages in peripheral blood and lack of the adhesive knobs in *P. vivax* suggestedlack of cytoadhesion and sequestration in deep capillaries. However, presence or absence of sequestration and cytoadhesion in *P. vivax* remains a dogma as few reports suggested cytoadhesion to spleen (del Portillo et al., 2004) and lungs (Anstey et al., 2007). In Field et al. (1963) proposed sequestration of *P. vivax,* as disappearance of schizonts from the peripheral blood of *P. vivax* infected patient was observed (Field et al., 1963). Another recent report, showed absence or very low presence of schizonts in peripheral blood (Lopes et al., 2014). In addition, accumulation of schizont and gametocytes in bone marrow was also observed (Baro et al., 2017). The extracellular vesicles (EVs) present in the plasma of *P. vivax* infected patients contain parasite protein that facilitate the adhesion of infected reticulocytes to human spleen fibroblasts (hSFs). Human spleen fibroblast uptakes the *P. vivax* infected EVs that induces the expression of ICAM1 after the binding of transcription factor NF-kB (NF-kB translocates from cytosol to nucleus). Increased expression of ICAM-1 on hSFs membrane facilitates reticulocytes sequestration where parasites cytoadhere and multiply in spleen microvasculature (Toda et al., 2020). This report further supports the hypothesis of *P. vivax* cytoadherence and sequestration.

Nevertheless, this Plasmodium species contains circular gametocytes similar to most of the Plasmodium genus except *Plasmodium falciparum* and *Plasmodium reichenowi* (Mueller et al., 2009) which contain elongated gametocytes. Another characteristic that has clinical significance is that the *P. vivax* gametocytes appear early (after 4 days of infection) and can be observed in the peripheral blood before the onset of clinical manifestation (Mueller et al., 2009). Report suggests gametocyte production may start with the first generation of merozoites (McKenzie et al., 2002). However, it remains unclear when the sexual commitment does start.

## *Plasmodium vivax in vitro* culture

Continuous long-term *in vitro* culture (*Plasmodium falciparum*, *Plasmodium knowlesi*, and *Plasmodium cynomolgi*) has proved to be a crucial tool for understanding the parasite’s life cycle and invasion process in detail (Trager and Jensen, 1976; Kocken et al., 2002; Grüring et al., 2014; Chua et al., 2019). However, such a system for long-term *in vitro* culture does not exist for *Plasmodium vivax*. Developing a method that allows the continuous growth and propagation of *Plasmodium vivax* would provide us new research opportunities to explore the molecular details of *Plasmodium vivax* malaria. The limitations and research breaches of *P. vivax* blood stage culture have been discussed here in detail. In addition, alternate research avenues being developed and explored by vivax malariologists, have also been discussed.

**Figure 2:**
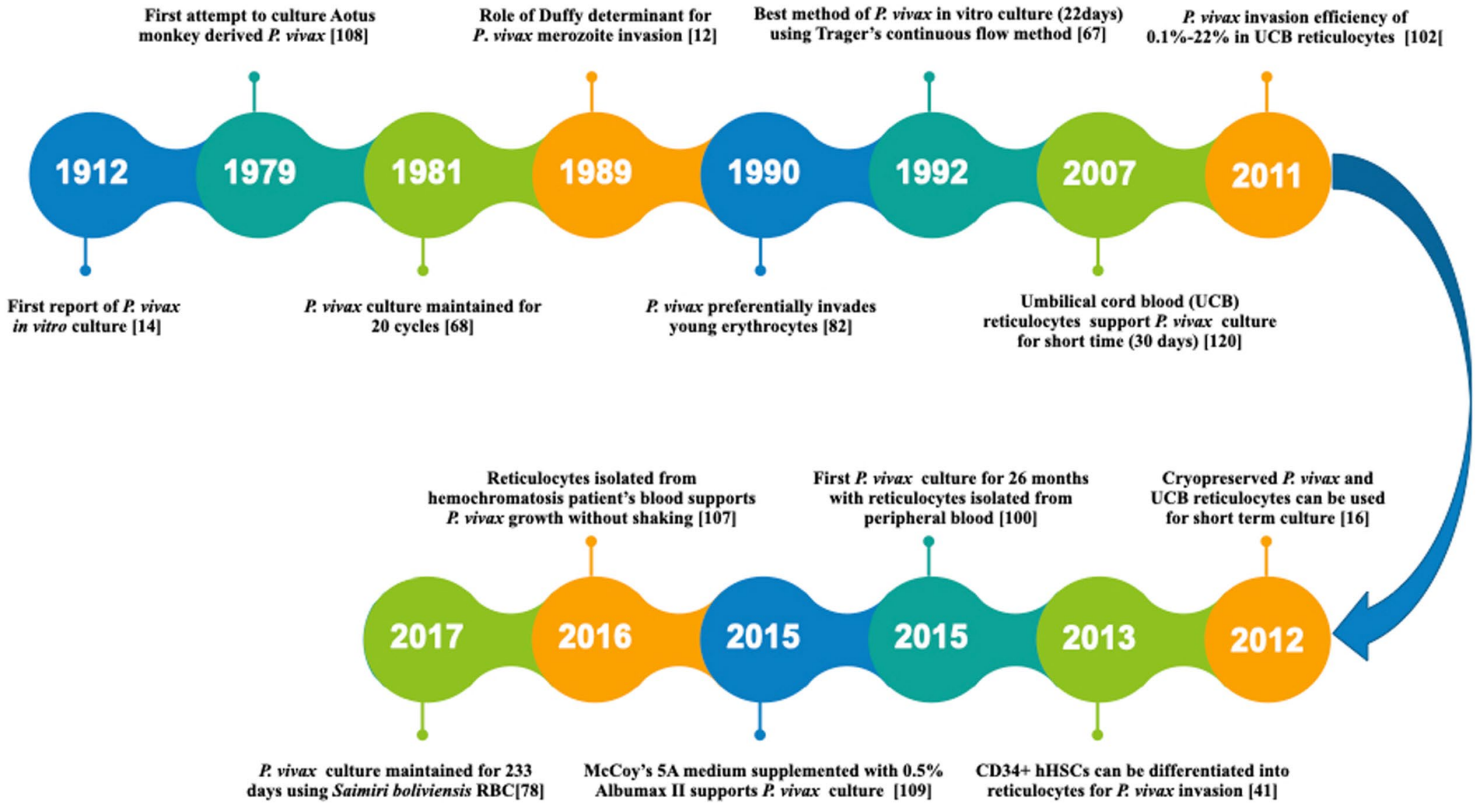
A timeline representing the major developments towards *P. vivax in vitro* culture. [14]: Bass and Jones (1912) [108]: Siddiqui (1979) [68]: Larrouy et al. (1981) [12]: Barnwell et al. (1989) [82]: Mons (1990) [67]: Lanners (1992) [120]: Udomsangpetch et al. (2007) [102]: Russell et al. (2011) [16]: Borlon et al. (2012) [41]: Fernandez-Becerra et al. (2013) [100]: Roobsoong et al. (2015) [109]: Singh et al. (2015) [107]: Shaw-Saliba et al. (2016) [78]: Mehlotra et al. (2017).

The first successful *P. vivax in vitro* culture was reported in 1912 (Bass and Johns, 1912). However, the detailed protocol for propagating this parasite was established only by the mid of 1970s (Trager and Jensen, 1976). Since then, several modifications have been attempted to improve the culture conditions by modifying the culture media, parasite origin and reticulocyte origin as reviewed recently in detail by others (Bermudez et al., 2018; Thomson-Luque et al., 2019). A timeline representing the major developments towards *P. vivax* culture optimization has been shown in Figure 2. In a major development, *P. vivax* culture was maintained continuously for 233 days (0.1% parasitemia) using new world monkey (*Saimiri boliviensis*) blood along with DMEM (Dulbecco’s Modified Eagle Medium), L-glutamine, HEPES (4-(2-hydroxyethyl)-1-piperazineethanesulfonic acid) and hypoxanthine (Mehlotra et al., 2017). This study leads to the belief that long term culture of *P. vivax* culture is not impossible anymore. However, further validation is required with human peripheral blood.

Despite continuous efforts to culture *P. vivax*, it remains difficult primarily because of the progressive loss of parasite’s invasion efficiency, i.e., the percentage of infective merozoites that are able to invade and establish infection in reticulocytes (Thomson-Luque et al., 2017; Bermudez et al., 2018; Gunalan et al., 2020). However, the biological basis of the loss of parasites’ invasion efficiency is far from clear. A thorough analysis is needed to be done considering host, parasite and environmental factors. Here, an attempt has been made to understand all the three factors in detail.

### Host factors

*Plasmodium vivax* requires host reticulocytes for its growth and propagation (Bass and Johns, 1912; Mons, 1990). Reticulocytes are precursor red blood cells, slightly larger (10–15 mm) than the mature red cells (6–8 mm) and having reticular network of residual RNA. Heilmeyer and Westhauser observed diverse nature of reticulocytes in supravital stained blood smear and classified them in four classes (I–IV). A very young reticulocyte without nucleus having dense coherent mass of RNA along with other organelles is called as Heilmeyer class I (HCI) reticulocyte. Further mature reticulocytes having reticular network of RNA instead of original dense mass are called as HCII reticulocyte. HCIII reticulocytes have lesser amount of dispersed RNA. The most mature reticulocytes having dispersed remnants of RNA are called as HCIV reticulocytes (Heilmeyer and Westhäuser, 1932). Whereas HCI and HCII early stage reticulocytes are present in bone marrow, HCIII reticulocytes are mostly found the in bone marrow and HCIV are the most common form of reticulocytes present in the peripheral blood (Seip, 1953). Early stage reticulocytes are large, rigid, and irregular in shape but mature reticulocytes become smaller, and biconcave in shape (Malleret et al., 2013). Reticulocytes represent only 1%–2% of circulating human red blood cells and have a short life span of ~4 days:3 days in bone marrow and 24 h in circulation (Kaufman, 2017).

The maturation of reticulocytes is indicated by decreased expression of transferrin receptor (CD71) (Kono et al., 2009; Malleret et al., 2013). The majority of immature CD71^+^ reticulocytes (HC0, I, II, and III) are restricted to the bone marrow and remain more invasive to *P. vivax* as compared to the CD71^−^ cells (HCIV reticulocytes or erythrocytes) and minor population of CD71^+^ reticulocytes present in the peripheral blood (Malleret et al., 2015). CD71 has been shown to be a critical reticulocyte receptor for invasion by *P. vivax* through the parasite ligand, PvRBP2b (Gruszczyk et al., 2018) along with the *P. vivax* Duffy-binding protein/Duffy antigen receptor for chemokines channel (Kanjee et al., 2021). The preferential invasion in CD71^+^reticulocytes may be because immature reticulocytes are rich in iron and other metabolites (Thomson-Luque and Bautista, 2021) which are required for parasite maturation. Immature reticulocytes synthesise haemoglobin and ~20%–30% of total haemoglobin is synthesised at this stage of the red blood cells (Riley et al., 2001). Nevertheless, Malleret et al. revealed that *P. vivax* induces rapid maturation of reticulocytes *ex vivo*, may be to protect itself from the continuous deformations of immature reticulocytes (Malleret et al., 2015). Later, Lim et al. confirmed that red blood cell maturation does not happen so rapidly *in vivo* as early stage parasites are found to be present always in reticulocytes (Lim et al., 2016). However, understanding the specific crosstalk between maturing *P. vivax* and maturing reticulocytes may have impact on long term *in vitro* cultivation of *P. vivax*.

CD71 (transferrin receptor1) being an iron importer protein, brings transferrin bound iron inside the reticulocytes (Jandl et al., 1959). Interestingly, reticulocytes isolated from haemochromatosis patients represented 14%–17% of total red blood cells compared to 6.9%–7.9% from umbilical cord reticulocytes (Udomsangpetch et al., 2007). Haemochromatosis is an inherited disorder characterised by excess iron accumulation in tissues and organs (Andrews et al., 1999) andphlebotomy is an effective treatment of hemochromatosis as it removes excess iron present in blood. Post-phlebotomy, haemoglobin concentration and serum iron level decreases and bone-marrow becomes highly active leading to increased reticulocytosis to compensate red blood cell loss and a slower reticulocyte maturation rates (Conrad et al., 1962; Gildersleeve et al., 1985; Feeney et al., 2005). Further, there are reports showing severe anaemia in case of *P. vivax* infection despite having low parasitic burden (Mohapatra et al., 2002; Collins et al., 2003; Kenangalem et al., 2016; Punnath et al., 2020). One of the reasons could be that *P. vivax* may require more iron for its growth and survival which results in host iron scarcity and ultimately anaemia. In one of the most successful attempts to culture *P. vivax* till date, Golenda et al. used haemochromatosis patient’s reticulocytes and observed almost 2-fold increase in parasitemia after each cycle which was higher compared to other methods (Panichakul et al., 2007; Noulin et al., 2012, 2014). According to a recent report by Shaw-Saliba et al., *P. vivax* showed significant preference for reticulocytes isolated from fresh haemochromatosis patients’ blood. They achieved a maximum of 1.8% parasitemia using McCoy’s 5A media supplemented with 20% AB+ heat inactivated serum, 6% haematocrit in a candle jar (~18% oxygen; Shaw-Saliba et al., 2016). Noteworthy, Golenda et al. achieved 5-fold increase in the number of reticulocytes after centrifugation steps whereas Shaw-Saliba et al. could obtain a maximum of 3-fold increase in reticulocyte from starting hemochromatosis blood. It could be because of differences in reticulocyte count which may be due to different treatment procedure, different number of bleeds and phlebotomy of hemochrombtosis patients’ (Shaw-Saliba et al., 2016). Altogether it seems that reticulocytes isolated from haemochromatosis patient blood remains more invasive to *P. vivax* infection compared to other blood sources, possibly because of the abundance of iron and more number of CD71+ reticulocytes in haemochromatosis blood (Conrad et al., 1962; Gildersleeve et al., 1985; Feeney et al., 2005).

A recent report by Gunalan et al. suggested that oxidative stress due to higher (as compared to that in bone marrow) ambient O_2_ during various culture conditions/steps could have negative effect on *P. vivax* growth. Further, reticulocytes’ high glucose-6-phosphate dehydrogenase (G6PD) content (that reduces as RBCs mature) counteracts this oxidative stress and hence offer a congenial micro-environment for *P. vivax* growth (Luzzatto, 2006; Wilson et al., 2016; Gunalan et al., 2020). However, supplementation of antioxidants like glutathione, ascorbic acid, selenium, pyruvate, and lipoic acid does not improve *P. vivax* growth. It is further suggested that a combination of antioxidants along with other culture-condition modifications, including culture in hypoxia-mimicking conditions and use of more membrane-permeable antioxidant compounds, might help in relieving the oxidative stress present inside the reticulocytes (Gunalan et al., 2020). It is noteworthy that iron (Fe^+2^) also acts as pro-oxidant and can induce cellular oxidative stress (Puntarulo, 2005; Colpo et al., 2008). It would be interesting to examine if iron chelators can be used to mitigate the cellular oxidative stress that was induced by iron (Fe^+2^). Adding to the list of antioxidants that were described by Gunalan et al., deferoxamine, deferiprone and deferasirox may also be explored.

Clark et al. examined the structural integrity of *Plasmodium vivax* infected reticulocytes using a flowcytometry-based assay that measured the osmotic stability of reticulocytes. It was observed that immature CD71^+^ reticulocytes were osmotically most stable amongst other mature stages of CD71^−^ reticulocytes and normocytes. *P. vivax* infected reticulocytes were less stable compared to uninfected CD71^+^ reticulocytes, suggesting that *P. vivax* infection compromises the reticulocyte stability. In addition, it was observed that *P. vivax*-infected reticulocytes were more instability as compared *P. falciparum-*infected normocytes. Interestingly, reticulocytes acquired after *in vitro* differentiation of stem cells have been shown to be less stable compared to *ex vivo* reticulocytes (Clark et al., 2021). This could be one of the reasons behind the observation that *P. vivax* better propagates in *ex vivo* culture. It thus implies that the reagents that can increase the osmotic stability of reticulocytes *in vitro*, would have impact on long term culture of *Plasmodium vivax* and, to cite an example, cholesterol has been shown to increase the osmotic stability of reticulocytes present in *in vitro* culture (Clark et al., 2021).

Rapid maturation of reticulocytes also turns out to be a restrictive factor when using these cells for assays which take a longer time to reach to the assay endpoint. Borlon et al. analysed the probability of cryopreserving reticulocytes which were obtained from umbilical cord blood. Reticulocytes were isolated as per the earlier established protocol (Russell et al., 2011). Subsequently, cells were homogenised in 20% glycerol and preserved in liquid nitrogen. It was shown that liquid nitrogen preservation did not affect reticulocytes stability and viability as they matured normally and supported parasite invasion (Borlon et al., 2012). Further, Noulin et al. showed that erythrocytes obtained from haematopoietic stem cells could be cryopreserved for 1 year without disturbing the receptors present on the cell membrane. Two different media containing different preservative agents were used for reticulocytes cryopreservation and both were successful. The first consisted of 28% glycerol, 3% sorbitol and 0.9% sodium chloride, whereas the other contained 10% dimethyl sulfoxide (DMSO) and 40% fetal calf serum (Noulin et al., 2012).

### Parasite factors

Despite the continuous supply of fresh reticulocytes, *P. vivax* culture remains challenging due to a variety of parasite-associated variables, including inefficient invasion after few rounds of asexual intra-erythrocytic cycles, lack of maturation and egress. The incompetent parasite invasion to reticulocytes under *in vitro* condition remains one of the biggest hurdles (Thomson-Luque et al., 2017). Thomson-Luque has enlisted some of the problems observed during *P. vivax in vitro* culture: pyknotic rings, lack of Schuffner dots, delayed trophozoite formation, presence of gametocytes instead of blood stages, appearance of crisis forms of parasites (broken pieces of parasites or nutrient deficient unhealthy parasites; Thomson-Luque et al., 2017). Nonetheless, decrease in parasitemia was observed mostly at the trophozoite to schizont transition state. The reason behind this enigma remains unexplored. However, lack of a favourable environment could be one of the factors.

*Plasmodium vivax* isolates are genetically divergent in nature as each isolate having different level of reticulocyte preference. A study by Lim et al. using Indian *P. vivax* isolates showed that some of the *P. vivax* strains have less predilection towards reticulocytes in contrast to the reticulocyte restricted nature of the species, in general. Strains which have preference for reticulocytes, had more number of schizonts than gametocytes, suggested invasion to young reticulocyte helps in schizont development (Lim et al., 2016). In another study, Russell et al. examined invasion efficiency of the parasite using 85 clinical isolates of *P. vivax* from Thailand-Myanmar border. A high level of variation was observed in invasion efficiencies amongst all the *P. vivax* clinical isolates. However, invasion efficiency remains constant when a specific isolate was incubated with reticulocytes isolated from different ABO blood group donors. Altogether, it was observed that ~86% of the total variance in invasion efficiency was dependent on the parasite isolate type. Host blood group accounted for only 0.17% of total variation in invasion efficiency (Russell et al., 2011).

Both of the above mentioned studies indicate that each *P. vivax* human isolates has its own characteristics and this creates variations in invasion. Further, the unique characteristics of each isolate could be attributed to hitherto unknown genetic factors. These genetic determinants might regulate individual parasite isolate at transcriptomic, genomic or epigenomic levels. In future, the determinant that regulates the reticulocytes invasion is expected to have impact on long term blood stage culture of *P. vivax*.

Regular parasite-based invasion assays measure the average parasitemia from a group of parasites, assuming the average parasitemia is representative of each parasite’s invasion. However, in doing so a particular subpopulation of parasite may not be counted whose behaviour is different from rest of the parasites. Therefore, single parasite culture should be performed as standardised by others (Miao and Cui, 2011) and its growth pattern should be monitored. Fluorescence activated cell sorting (FACS) based single parasite isolation from complex parasite population could provide insights on the genetic variation at individual parasite level.

### Environmental factors

The third most important factor involved with *P. vivax in vitro* culture is the culturing medium (the environment present around the parasite). The detail regarding the culture media formulation for *P. vivax in vitro* culture, has already been reviewed by others in detail (Bermudez et al., 2018; Thomson-Luque et al., 2019). Therefore, it has not been discussed here. Moreover, McCoy’s 5A medium with human serum is the most commonly used formulation for *P. vivax* propagation in *in vitro* condition. However, McCoy’s 5A formulation has been reported to reduce *P. vivax* parasites’ viability after 48 h of intraerythrocytic cycle (Borlon et al., 2012). Recent observation by Rengel et al. show that Iscove’s modified Dulbecco’s medium (IMDM) supports parasites’ intraerythrocytic growth better compared to other formulations (Rangel et al., 2018). However, no significant difference was observed in transcriptomic signature distinguishing parasites grown in IMDM versus other media. Nonetheless, *Plasmodium vivax* parasites’ density does decline over time even with IMDM and it represents a major obstacle to sustained *in vitro* culture of this parasite.

FACS-assisted single cell culture of *P. vivax* infected reticulocytes can be performed in order to explore the inhibitory effect of one parasite on the growth and survival of neighbouring parasites and subsequently parasite invasion, maturation, egress can be monitored. Further, several reports suggest that each *P. vivax* isolates has its unique behaviour in *in vitro* condition. Isolate specific metabolomics study may help in understanding the differences in metabolic pathways operational in *in vitro* condition. The metabolomics exploration study would aid in optimization of culture media and growth factors required for each isolate. Nevertheless, Rengel et al. attempted similar study, in which 1000 FACS-purified parasites from viable cryopreserved patient isolates were cultured in different media formulations and transcriptome analysis was performed at various parasite development stages. Further, it was observed that culture media has not much effect on parasites’ transcriptional profile (Rangel et al., 2020). The lack of parasites’ transcriptional response in different culture media might indicate the altered role of host cells (red blood cells) itself. However, more studies required in order to explore the role of different media on Parasites’ survival. In addition, single-cell transcriptomics approaches would help further in characterising the variation between the parasites within an isolate.

Altogether, the parasite, environment and host factors appear to the determining factors for *Plasmodium vivax in vitro* culture, either singly or in combination. This includes the role of parasite genes and gene expression in maintaining parasite invasion and parasite biomass during *in vitro* cultures. Further, *Plasmodium vivax* parasites lack molecular and genetic determinants required for drug selection and identification of biomarkers. Development of *P. vivax* transgenic parasites has suffered a stback from lack of an *in vitro* culture system together with low transfection efficiency and lack of selection markers. Despite these challenges, few attempts have been made in order edit this parasite at molecular level.

## *Plasmodium vivax* blood stage transfection

Despite the development of emerging methodologies for genome editing, the genetic manipulation of *Plasmodium vivax* still remains poorly attempted. The lack of efforts to genetically manipulate this parasite is mostly attributed to the absence of long term *in vitro* culture and a poor availability of molecular tools to genetically manipulate the parasite. This is because stable gene manipulation generally requires a continuous maintenance of 3–4 weeks of asexual propagation and by that time *P. vivax* may not survive *in vitro* conditions due to the reasons mentioned above. In case of stable transfection, plasmid DNA initially replicates episomally in the cytoplasm and subsequently during drug selection (for 2–3 weeks), it integrates into the genome by single or double crossover homologous recombination and such successful transformants are further confirmed through Southern blotting (Crabb et al., 1997). In contrast, transient transfection can be performed easily with *P. vivax* as it requires only 48–96 h of *in vitro* maintenance after transfection (Crabb et al., 2004). However, a transgenic *P. vivax* line development and gene function analysis through knockout study requires stable transfection.

Another important factor associated with *Plasmodium vivax* transfection is the lack of gene regulatory elements required for *P. vivax* specific plasmid construction. *Plasmodium falciparum* histidine rich protein3 promoter and calmodulin promoter has been shown to work in *P. vivax* (Pfahler et al., 2006). Moraes Barros et al. were successful in using *P. vivax* calmodulin promoter for zinc finger nucleases (ZFN) expression in *P. vivax* (Moraes Barros et al., 2015). However, several attempts to characterise *P. vivax* promoter in *Plasmodium falciparum* blood stage culture remains unsuccessful (Azevedo and del Portillo, 2007). Nevertheless, for the first time *P. vivax* centromere from chromosome11 (*Pv*CEN) and promoter of heat shock protein 70 (*pv*hsp70) was shown to be active in *P. yoelii* (Thawnashom et al., 2019). These regulatory sequences can be utilised for *P. vivax* specific plasmid construction and gene manipulation in future.

*Plasmodium vivax* transfection would be a crucial step towards making the parasite more compliant to molecular inquest. Till date, only two independent attempts have been made to transfect *P. vivax* trophozoites. Pfahler et al. transiently transfected *P. vivax* blood stage parasites using Luciferase construct (Pfahler et al., 2006). *P. vivax* SalvadorI strain was used for transfection and parasites were isolated from infected splenectomised *Saimiri boliviensis* monkeys. The purified *P. vivax* infected trophozoites were mixed with Luciferase-containing plasmids and electroporated using a Gene Pulser Xcell™ electroporation system (BioRad). The transfected reporter genes were expressed despite being under the control of *P. falciparum* 5′ and 3′ regulatory elements. This indicated that the regulatory regions are conserved between both the species. In another major development in transfection technology, Morales Barros et al., stably transfected *P. vivax* Chesson strain using zinc-finger nucleases. A specific mutation (quadruple mutant) was introduced to the gene encoding *P. vivax* dihydrofolate reductase (*pvdhfr*) and it was selected using the drug pyrimethamine in *Saimiri boliviensis*. Blood samples were collected from *P. vivax* Chesson infected splenectomised *Saimiri boliviensis* monkey and infected erythrocytes were electroporated *ex vivo* using ZFN plasmid containing mutant *pvdhfr* sequence (Moraes Barros et al., 2015). Customised ZFNs can now be used for genetic manipulations in *P. vivax*. Further, mutant *pvdhfr* or human *dhfr* or other selectable markers can be used for selection in *P. vivax*. In absence of continuous *in vitro P. vivax* culture system, transfection and selection has been done in expensive nonhuman primates.

Transfection associated variables such as electroporation condition or *P. vivax* developmental stage to be transfected, has not been optimised due to the limited availability of *P. vivax* infected red blood cells (Pfahler et al., 2006). However, from the above two transfection study it appears that *P. vivax* infected blood containing all the developmental stages can be used for transfection unlike other Plasmodium species (Table 1) which prefer a specific stage. Anyway, a stage specific transfection needs to be done in order to explore the transfection efficiency of each developmental stage of *P. vivax*.

**Table 1:** Preferred blood stage for transfection in different Plasmodium species.

Plasmodium species	Preferred blood stage to be transfected
*P. falciparum*	Ring stage (Wu et al., 1996)
*P. knowlesi*	Mature schizont (van der Wel et al., 1997)
*P. cynomolgi*	Mature schizont (Kocken et al., 1999)
*P. berghei*	Mature schizont (de Koning-ward et al., 2000)
*P. vivax*	Trophozoite (Pfahler et al., 2006) and infected blood containing all the stages (Moraes Barros et al., 2015)

## Options to study *Plasmodium vivax* biology

### Reticulocyte producing cell lineage

Reticulocyte availability is the limiting factor in success of *P. vivax in vitro* culture. Therefore, there is need of a reticulocyte producing cell lineage that can supply uninterrupted, homogenous population of reticulocytes. To date, few attempts have been made to generate immortalised lines of human erythroid cells from haematopoietic stem/progenitor cells (Kurita et al., 2013; Huang et al., 2014), embryonic stem cells (Hirose et al., 2013) or induced pluripotent stem (iPS) cells (Kurita et al., 2013). However, the enucleation rate of iPS cells are less compared to embryonic stem cells. In addition, reticulocytes produced from embryonic stem cells contain fetal haemoglobin that may have adverse effect on *in vitro* culture. Hematopoetic stem cells isolated from bone marrow or peripheral blood represents a good alternative for reticulocyte line generation. However, bone marrow derived stem cells isolation remain invasive whereas stem cells derived from peripheral blood are easily accessible.

Recently, normal human erythroid cells has been used for making immortalised adult erythroid line (Trakarnsanga et al., 2017). Bristol Erythroid line Adult (BEL-A) is the first human immortalised adult erythroid line derived from adult bone marrow CD34+ cells that generates mature reticulocytes after enucleation. Morphologically and functionally BEL-A resembles that of *in vitro* cultured reticulocytes (Trakarnsanga et al., 2017). Further, BEL-C and BEL-P erythroid line have been generated from cord blood and peripheral blood CD34+ cells (Daniels et al., 2021). Both, BEL-C and BEL-P has been shown to recapitulate the characteristics of their parental cells in terms of global expression, differentiation profile and overall proteome profile. Moreover, generation of BEL-A, BEL-C and BEL-P lines have open a door to study host–parasite interaction and its aftereffects. Next, the suitability of these lines for *P. vivax* infection and *in vitro* culture can be examined.

### Humanised mice model

CD71 producing human red blood cells are the limiting factor for *P. vivax in vitro* culture. Luiza-Batista et al. (2022) attempted to develop a mice model having human erythropoiesis after engraftment of human haematopoietic stem and progenitor cells. This mice model supported *de novo P. vivax* growth and proliferation after 3-weeks of infection. The mouse strain called as HIS-HEry (Human Immune System-Human Erythrocyte) survived for 1 year and mimicked several key features of *P. vivax* infected humans including localisation of sexual stages of parasite to bone marrow, gemetocyte formation and transmission to Anopheles mosquitoes despite having low parasitaemia. Though the current model has opened new avenues in understanding *Plasmodium vivax* biology, due improvements are required, towards low number of circulating human RBCs and low number of sporozoite production after gametocyte transmission (Luiza-Batista et al., 2022). Notably, the low number of peripheral human RBCs attributes to their elimination by murine macrophages (Hu et al., 2011). Further, low number of peripheral RBCs restricts the numbers of gametocytes transmissible to mosquitoes and therefore, the current mice model requires further optimisation.

### Primate malaria model – *plasmodium cynomolgi*

*Plasmodium cynomolgi* is a relapsing primate malaria parasite and currently it is used as a model system for *P. vivax*. Both, phylogenetically and phenotypically, *P. cynomolgi* and *P. vivax* are quite close to each other (Tachibana et al., 2012). An improved genome sequence assembly of *P. cynomolgi* revealed 282 similar gene clusters between *P. cynomolgi* and *P. vivax,* this further strengthens that they are close related (Pasini et al., 2017) The blood stage culture of *P. cynomolgi* line derived from Berok strain, was recently developed by Chua et al. (2019). The availability of *P. cynomolgiin vitro* culture would open new avenues to *P. vivax* research as it can be utilised for high-throughput drug screening and genetic manipulation which remains unachieved for *P. vivax*. Successful *in vitro* propagation of *P. cynomolgi* Berok K4 line was obtained in six independent laboratories. Mature schizont formed after 46–48 h of infection with eight to 16 merozoites whereas gametocytes observed from day6 onwards and overall gametocytaemia remains low with 0.01%. Noteworthy, *P. cynomolgi* M and B strains failed several attempt to culture *in vitro* (Chua et al., 2019).

### *Plasmodium vivax* gene manipulation *via Plasmodium knowlesi*

*Plasmodium vivax* remains distinct from *P. knowlesi* in terms of hypnozoite formation and its preference for reticulocytes. However, the two parasites are phylogenetically closely related and dependent on Duffy binding proteins for host cell invasion (Adams et al., 1990; King et al., 2011). In absence of long-term *in vitro* culture, the *P. vivax* research is mostly constrained to recombinant protein assays, *ex vivo* studies, primate infection and controlled human malaria infection (Russell et al., 2011; Shakri et al., 2012; Payne et al., 2017). The adaptation of *P. knowlesi* to long-term culture (Butcher, 1979) provides a unique platform to explore the basic biology of *P. vivax*. Further, *P. knowlesi* can be cultured in human RBC with human serum without the need of rhesus monkey RBC and serum (Moon et al., 2013). The transfection efficiency achieved with this human-adapted *P. cynomolgi* line is 30%–40% which is 100,000 fold more than that of *P. falciparum* and surpasses that obtained with *P. berghei.* Because of the high transfection efficiency, the transgenic parasites can be observed in the first generation itself after transfection. However, the major drawback associated with this line is absence of gametocyte production (Moon et al., 2013).Moreover, Mohring et al. performed clustered regularly interspaced short palindromic repeats (CRISPR-Cas9) mediated genome editing in *P. knowlesi*. The key parameters involved in CRISPR-Cas9 mediated genome editing were examined and it emerged as a highly robust process with 100% successful transfection (Mohring et al., 2019). This will enable genome-wide systematic knockout or gene tagging in a *Plasmodium* parasite that can infect human.

### Passive merozoite “invasion” in reticulocytes

Despite standardising different aspects, maintaining a continuous *P. vivax in vitro* culture remains elusive. The lack of continuous *in vitro* culture is predominantly attributed to the inability of *P. vivax* merozoites to invade new reticulocytes (Russell et al., 2011; Borlon et al., 2012; Tantular et al., 2012). To overcome the hurdles of successful active merozoite invasion in to reticulocytes, merozoites could be passively introduced into the reticulocytes through electroporation (as in routine transfection procedures) or intracytoplasmic merozoite injection (akin to the procedure of intracytoplasmic sperm injection) and its after-effects be investigated. The size of merozoites is 1–1.5 μm and the diameter of red blood cells is in the range of 6–8 μm which makes it feasible, at least theoretically. It would be interesting to think of a direct delivery of free merozoites in to reticulocytes which can have significant impact on the success of *P. vivax in vitro* culture. However, the success of such intracytoplasmic delivery of free merozoites in red blood cells would entirely depend on the survival of injected merozoites lacking a parasitophorous vacuolar membrane (PVM). Isolation of viable merozoites has been well established in *Plasmodium knowlesi* (Dennis et al., 1975; Bannister and Mitchell, 1989; Lyth et al., 2018) and *Plasmodium falciparum* (Boyle et al., 2010) but it remains unattempted in *P. vivax*. The purified merozoites remain intact and retained their invasive capacity. The established protocol of merozoite isolation can be utilised for *P. vivax* merozoites isolation and reticulocyte invasion assays or for direct delivery in reticulocytes.

However, the direct delivery of merozoites in the reticulocyte cytoplasm may not form the PVM and subsequently the parasite may not survive. Although PVM is known to aid the transport of proteins from parasite cytoplasm to the host cell cytoplasm, nutrients uptake and waste excretion (Goldberg and Zimmerberg, 2020), PVM is also shown as non-essential for every intracellular apicomplexans; as some of the parasites belonging to the genus Theileria and Babesia can survive without PVM in the host cytoplasm (Lingelbach and Joiner, 1998; Shaw, 2003; Repnik et al., 2015). Further, the significance of PVM in Pasmodium parasites remains elusive (Goldberg and Zimmerberg, 2020). Merozoites intracytoplasmic injection in reticulocytes might open a door to understand the biology of Plasmodium PVM as well.

## Conclusion

In the last 10 decades, several attempts have been made to establish *P. vivax in vitro* culture with minor achievements. However, significant advancements have been made towards primate-adapted *P. vivax in vitro* culture. Continuous long-term maintenance of *P. vivax in vitro* growth with high(er) parasitemia using human isolates is still unachieved and requires due attention. The most enigmatic factor associated with *P. vivax in vitro* culture is the lack of reproducibility with the published protocol. Golenda et al. (1997) developed the most successful protocol to culture these parasites for 6–8 generation using hemochromatosis patients’ blood. Shaw-Saliba and colleagues reattempted to optimise Golendas’ protocol using *P. vivax* primate-adapted strain Sal-1 obtained from *Aotus lemurinus lemurinus.* However, the results of Golenda study was not replicated and the reason could be difference in young reticulocyte contents, iron levels or may be because of differences in strains of *P. vivax* used. Each human *P. vivax* isolate has its unique growth kinetics in *in vitro* system which it could be due to unique genetic makeup of individual isolates and this needs to be explored to improve the current efforts to sustain *in vitro P. vivax* cultures. Further investigations are required in order to address the variables associated with Golendas’ and other researcher’s study. Moreover, reticulocytes from haemochromatosis patients do support *P. vivax* growth *in vitro* may be because of abundance of iron in them, however, reticulocytes’ osmotic stability plays an important role in *P. vivax* infection that might impact long term *P. vivax in vitro* culture. The efforts should also include the possibilities of making an immortalised reticulocyte line from erythroid progenitor cells and some blue sky research using passive delivery of *P. vivax* merozoites into reticulocytes/RBCs.

With regards to transfection success stories, transient transfection has been achieved with *P. vivax* however, further optimisation is required using human isolates. Genetic manipulation in *P. vivax* has not been successfully attempted yet. Since lack of continuous culture system is a major limitation in advancement of transfection technologies in *P. vivax*, the use of alternate Plasmodium species (*P. cynomolgi* and *P. knowlesi*) that mimic some of the biology of *P. vivax* may be encouraged as facultative approaches, till an ideal *P. vivax* long term culture system is established.

## Author contributions

SK drafted and wrote the manuscript. AS conceptualised, designed, edited and reviewed the manuscript. All authors contributed to the article and approved the submitted version.

## Funding

The work was funded by Indian Council of Medical Research (ICMR), New Delhi in the form of Postdoctoral fellowship to Sanju Kumari (ICMR-PDF).

## Conflict of interest

The authors declare that the research was conducted in the absence of any commercial or financial relationships that could be construed as a potential conflict of interest.

## Publisher’s note

All claims expressed in this article are solely those of the authors and do not necessarily represent those of their affiliated organizations, or those of the publisher, the editors and the reviewers. Any product that may be evaluated in this article, or claim that may be made by its manufacturer, is not guaranteed or endorsed by the publisher.

## Abbreviations

CD71: Cluster of differentiation 71
CRISPR/Cas9: Clustered regularly interspaced short palindromic repeats/Crispr associated protein9
DMEM: Dulbecco’s medium eagle medium
DMSO: Dimethylsulfoxide
FACS: Flourescence activated cell sorting
G6PD: Glucose 6-phosphate dehydrogenase
HC: Heilmeyer’s class
HEPES: 4-(2-hydroxylethyl)-1-piperazineethanesulfonic acid
PV: Parasitophorous vacuole
*Pv*-IRBCs: *Plasmodium vivax*-infected red blood cells
PVM: Parasitophorous vacuolar membrane
*Pv*dhfr: *Plasmodium vivax* dihydrofolate reductase
RBC: Red blood cell
ZFN: Zinc finger nuclease
